# *Ulomoides dermestoides* as an Insect Pharmacological Resource of Antioxidant and Anti-Inflammatory Bioactive Substances: Chemical Basis, Mechanisms of Action, Pharmacological Evidence, and Translational Challenges

**DOI:** 10.3390/antiox15070849

**Published:** 2026-07-05

**Authors:** Tianzi Wang, Wenling Shi, Xingyue Song, Jinglei Huang, Youqing Cheng, Xiaofan Zhang, Wei Xie, Guoqing Wan

**Affiliations:** 1 School of Chemistry and Chemical Engineering, Shanghai University of Engineering Science, Shanghai 201620, China; wtz5254011302@163.com (T.W.); xingyue_song@163.com (X.S.); zhangxf@sumhs.edu.cn (X.Z.); 2 Digital and Intelligent Empowerment Biomedical Innovation Center, School of Pharmacy, Shanghai University of Medicine and Health Sciences, Shanghai 201318, China; 18402109972@163.com (W.S.); 13764029631@163.com (J.H.); 18844987215@163.com (Y.C.); 3 Shanghai Key Laboratory of Molecular Imaging, Shanghai University of Medicine and Health Sciences, Shanghai 201318, China

**Keywords:** *Ulomoides dermestoides*, natural products, antioxidant, anti-inflammatory, UGP2A, TLR4/MyD88

## Abstract

*Ulomoides dermestoides* (Yangchong) is a tenebrionid beetle used in traditional medicine across Asia and Latin America. While crude extracts show effects on inflammation, oxidative stress, and other conditions, systematic integration of its bioactive substances, mechanisms, and translational potential is lacking. This review consolidates its chemical basis, comprising volatile benzoquinones, terpenes, and alkenes, alongside non-volatile fatty acids, proteins (antioxidant enzymes, glycoproteins), and phenolics. Pharmacological evidence indicates multi-target modulation of reactive oxygen species (ROS), cytokines, leukocyte recruitment, endothelial activation, and thromboinflammation. Recent advances include proteomic identification of antioxidant protein complexes, neuroprotection in a Parkinson’s disease model, chromosome-level genome assembly, and isolation of the UDP-glucose pyrophosphorylase 2a (UGP2A) glycoprotein, which alleviates thrombosis partly via toll-like receptor 4/myeloid differentiation primary response 88 (TLR4/MyD88)-mediated endothelial anti-inflammatory effects. However, most evidence remains preclinical, relying on non-standardized crude extracts, and benzoquinone-containing fractions display potential cytotoxicity and genotoxicity. Future research should integrate bioassay-guided isolation, structural characterization, multi-omics, pharmacokinetic/pharmacodynamic (PK/PD) analysis, standardized quality markers, and rigorous safety evaluation to transform *U. dermestoides* from an empirical insect-derived medicinal resource into a scientifically validated source of preclinical antioxidant and anti-inflammatory candidate substances.

## 1. Introduction

Natural products remain a vital source for discovering antioxidant and anti-inflammatory bioactive substances, as oxidative stress and chronic inflammation represent common pathological mechanisms in metabolic diseases, neurodegenerative disorders, vascular injury, tumor-associated microenvironments, and aging-related tissue damage [1,2,3,4]. Compared with plants and marine organisms, medicinal insects have received relatively less systematic attention; however, insects harbor chemically diverse small molecules, peptides, polysaccharides, lipids, enzymes, and defensive secretions produced by evolutionarily conserved stress response systems [5,6,7]. Therefore, insect pharmacology can establish a yet underexplored bridge between traditional animal-derived medicines, food-derived bioactive substances, and modern natural product pharmacology.

*Ulomoides dermestoides* (Fairmaire, 1893) (Coleoptera: Tenebrionidae), also reported in the literature under the names *Palembus dermestoides* and *Martianus dermestoides* [8,9], is commonly known as “Jiulongchong” or “Yangchong” in Chinese folk medicine, where it has traditionally been used to promote blood circulation, warm the spleen and stomach, relieve pain, and ameliorate weakness [9]. In Latin American folk practices, this insect has also been employed for respiratory diseases, inflammation, diabetes, Parkinson’s disease, and cancer-related symptoms [8,10]. It is important to emphasize that these traditional claims represent empirical observations without controlled clinical validation. They should be viewed as ethnopharmacological leads that justify systematic scientific investigation, not as evidence of clinical efficacy or safety. Although these traditional claims should not be accepted uncritically, they explain why *U. dermestoides* has continued to attract pharmacological interest in models related to inflammation, metabolism, vascular, gastrointestinal, and tumor conditions.

From a chemical perspective, *U. dermestoides* contains diverse classes of potentially bioactive substances. Volatilomic analyses have revealed that its defensive secretions are rich in benzoquinones (e.g., methyl-1,4-benzoquinone, ethyl-1,4-benzoquinone), terpenes (e.g., limonene, α-pinene), and long-chain alkenes (e.g., 1-pentadecene, 1-tridecene) [8,11]. Moreover, its cuticular hydrocarbon composition has been systematically characterized, providing a basis for chemotaxonomy and defensive chemical communication [11]. Analysis of aqueous extracts and essential oils has further identified fatty acids (e.g., palmitic acid, linoleic acid, oleic acid), phenolic compounds, alcohols, aldehydes, and alkanes, among other non-volatile constituents [8,12]. In an aqueous extract obtained by electro-pulse plasma dynamic extraction (EPDE), proteomic analysis identified antioxidant and stress-related proteins, including superoxide dismutase (SOD), peroxiredoxin, catalase, and heat shock proteins (HSP60, HSP70) [13,14]. Together, these chemical and proteomic studies indicate that *U. dermestoides* possesses both small-molecule antioxidants and macromolecular enzymatic antioxidant systems, forming a multi-layered basis of bioactive substances.

Regarding pharmacological activities, current evidence supports multi-target antioxidant and anti-inflammatory effects of *U. dermestoides* extracts or fractions. In vitro chemiluminescence assays showed that the antioxidant activity of *U. dermestoides* aqueous extract was equivalent to 0.2 mM Trolox, a water-soluble vitamin E analog, and this activity was attributed to the synergy between protein components (e.g., SOD, catalase) and non-protein components (e.g., ethylhydroquinone, phenolic compounds) [14]. In in vivo models, the aqueous extract significantly extended the mean lifespan of *Caenorhabditis elegans* under both normal and paraquat-induced oxidative stress conditions [14]. The lipid fraction of *U. dermestoides* exhibited hypoglycemic effects, increased insulin secretion, protected pancreatic and hepatic histoarchitecture, and upregulated mRNA expression of peroxisome proliferator-activated receptor gamma (PPARγ) and glucose transporter type 4 (GLUT4) in 3T3-L1 adipocytes in streptozotocin-induced diabetic mice [12]. In another study, the aqueous extract inhibited leukocyte recruitment and inflammatory exudation and modulated lymphocyte proliferation in a carrageenan-induced rat pleurisy model, further supporting its anti-inflammatory and immunomodulatory activities [15]. Additionally, *U. dermestoides* volatiles and essential oils were shown to possess repellent activity against stored-product pests [16], while whole-insect extracts concurrently exhibited certain cytotoxicity in anti-inflammatory assays, suggesting the possible coexistence of bioactive and toxic constituents [15]. Benzoquinones from its defensive secretions displayed cytotoxicity and genotoxicity toward A549 lung cancer cells [10]. These studies imply that the antioxidant and anti-inflammatory effects of *U. dermestoides* may involve multiple molecular mechanisms, including ROS scavenging, modulation of nuclear transcription factors, and improvement in insulin sensitivity.

Despite these advances, research on *U. dermestoides* still faces several issues and challenges. Most pharmacological evidence relies on crude extracts, and systematic isolation, structural characterization, and dose–response relationship studies of single bioactive substances are lacking. Second, in vivo pharmacokinetic (PK) and pharmacodynamic (PD) data for the bioactive constituents are virtually absent, and bioavailability and its influencing factors remain unevaluated. In terms of safety, although certain fractions such as lipids have shown good tolerability in animal studies [12], benzoquinone-containing fractions have been reported to exhibit cytotoxicity and genotoxicity [10,17], indicating that rigorous safety assessment is indispensable before developmental applications. Finally, sample heterogeneity arising from different origins, rearing conditions, and extraction methods hinders cross-study comparisons and reproducibility, underscoring the urgent need for standardization.

This review distinguishes itself from previous publications by providing, for the first time, a comprehensive integration of chemical, pharmacological, omics, and translational evidence for *U. dermestoides* within a unified framework. Unlike prior reviews that focused on isolated aspects such as traditional use, volatile chemistry, or specific pharmacological activities, our synthesis bridges the gap between chemical characterization (volatile benzoquinones, lipids, proteins, glycoproteins) and molecular mechanisms (SOD-CAT-HSP synergistic network, TLR4/MyD88/NF-κB signaling, thromboinflammation), while systematically addressing translational barriers (raw material heterogeneity, safety assessment, formulation challenges) that have hindered development. Furthermore, we present the first comprehensive integration of genomic evidence from the recently published chromosome-level genome assembly with proteomic and pharmacological data, proposing a multi-layered quality control framework that links gene expression to enzymatic activity and bioactivity. This integrated approach provides a conceptual roadmap for transforming *U. dermestoides* from an empirically used insect remedy into a scientifically validated source of bioactive substances with defined mechanisms and quality markers.

Several recent breakthroughs have provided new tools and directions for in-depth research on *U. dermestoides*. A chromosome-level genome assembly has been successfully completed, laying a genetic foundation for identifying genes encoding antioxidant- and anti-inflammation-related enzymes and metabolic pathways [18]. Combined proteomics and metabolomics strategies have enabled the first identification of antioxidant protein complexes from *U. dermestoides* larvae [14,19], and larval aqueous extracts have demonstrated neuroprotective effects in a paraquat-induced Parkinson’s disease-like model [20]. In 2026, a study isolated and characterized for the first time a glycoprotein designated UGP2A from *U. dermestoides*, which exhibits antithrombotic and endothelial anti-inflammatory activities partially dependent on the TLR4/MyD88 signaling pathway [21]. These advances indicate that the field is gradually transitioning from pharmacodynamic descriptions of crude extracts toward molecular mechanism elucidation and targeted isolation of active constituents.

### Literature Search Strategy

This review was conducted as a narrative synthesis of the available literature on the chemical composition, pharmacological activities, and translational potential of *Ulomoides dermestoides*. The following electronic databases were searched: PubMed, Scopus, Web of Science, Google Scholar, and China National Knowledge Infrastructure (CNKI) for both English and Chinese publications. The search period covered January 2000 to May 2026, with no language restrictions imposed. The following search terms and Boolean combinations were employed: (“*Ulomoides dermestoides*” OR “*Palembus dermestoides*” OR “*Martianus dermestoides*” OR “Jiulongchong” OR “Yangchong”) AND (“antioxidant” OR “anti-inflammatory” OR “chemical composition” OR “benzoquinone” OR “fatty acid” OR “protein” OR “glycoprotein” OR “pharmacology” OR “toxicity” OR “safety” OR “genome” OR “proteomics”).

Inclusion criteria comprised original research articles, reviews, and case reports; studies reporting chemical characterization, pharmacological activities (antioxidant, anti-inflammatory, metabolic regulation, vascular protection, neuroprotection), toxicological/safety data, or omics analyses (genomics, proteomics, volatilomics) of *U. dermestoides*; and studies published in peer-reviewed journals. Exclusion criteria included conference abstracts without full text, studies not directly related to *U. dermestoides*, studies with insufficient methodological detail for critical evaluation, and duplicate publications. A narrative synthesis approach was adopted rather than a meta-analysis. For each major pharmacological activity, we explicitly distinguished between evidence derived from crude extracts, evidence from isolated/purified compounds, and evidence from computational predictions.

## 2. Chemical and Macromolecular Basis of Bioactivity

**Figure 1:**
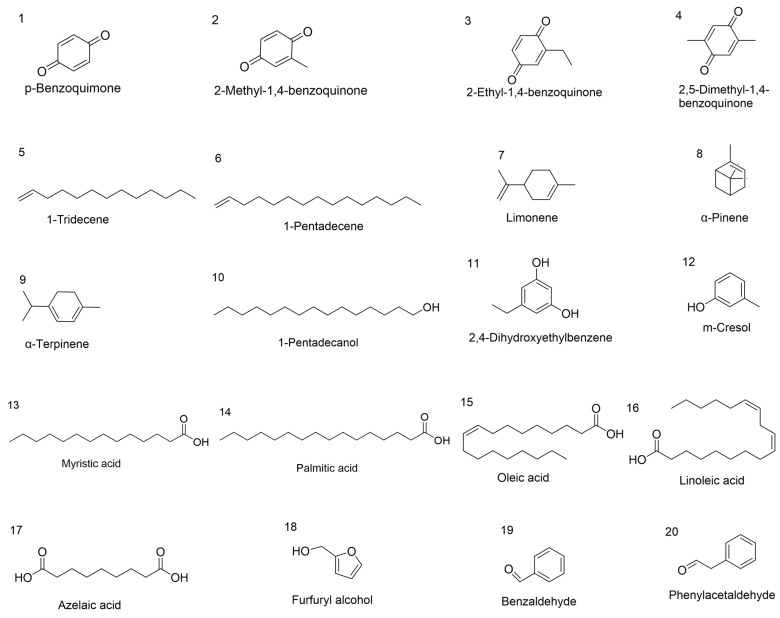
Representative chemical structures of compounds associated with *Ulomoides dermestoides* extracts, defensive secretions, or essential oils/volatilome. The compounds are numbered and labeled directly in the figure. Literature sources for each chemical class: benzoquinones (compounds **1**–**4**) and 1-pentadecanol (compound **10**) [8]; 2,5-dimethyl-1,4-benzoquinone and phenolics (compounds **4**, **11**, **12**) [18]; alkenes and terpenes (compounds **5**–**9**) [8,22]; fatty acids (compounds **13**–**16**) [12]; azelaic acid (compound **17**) [23,24,25]; and furfuryl alcohol, benzaldehyde, and phenylacetaldehyde (compounds **18**–**20**) [26].

The chemical space of *Ulomoides dermestoides* is diverse and rich, encompassing volatile defensive compounds, lipids and fatty acids, phenolics and related metabolites, proteins and peptides, enzymes and stress proteins, glycoproteins, and structural carbohydrates such as chitin. Different chemical classes differ markedly in extraction methods, stability, bioavailability, and safety profiles; understanding this chemical basis is a prerequisite for evaluating their pharmacological activity and developmental potential. Representative chemical structures of the major compound classes identified in *U. dermestoides* are shown in Figure 1.

### 2.1. Volatile Benzoquinones, Alkenes, and Terpenes

Benzoquinone-rich defensive secretions represent one of the most characteristic chemical attributes of *Ulomoides dermestoides* and related tenebrionid beetles. Early capillary gas chromatography analyses identified MBQ, EBQ, 1-tridecene, 1-pentadecene, and limonene as major components of the defensive volatiles of *U. dermestoides* [11,27,28,29]. Methyl-1,4-benzoquinone (MBQ) and ethyl-1,4-benzoquinone (EBQ) together account for over 90% of the total volatiles released under stress [11]. These benzoquinones are ubiquitous among Tenebrionidae and often co-occur with 1-alkenes and monoterpene hydrocarbons to form defensive blends [28,30].

Cázares-Samaniego et al. significantly expanded the chemical knowledge of this field by employing Headspace solid-phase microextraction (HS-SPME) fibers of different polarities and simulated gastrointestinal stress conditions [8]. Terpenes, quinones, alkenes, and aromatic compounds were identified in the headspace, while a wealth of terpenes, carboxylic acid derivatives, alkenes, alkanes, alkyl disulfides, alcohols, aldehydes, and quinones were detected in the essential oil, among which 171 compounds were reported for the first time in this insect [8]. The volatile chemical composition is dynamic: the relative abundances of quinones, limonene, pinene, and 1-pentadecene changed over time and under simulated digestive stress conditions, suggesting that the ingestion process may alter the profile of accessible metabolites [8,31]. Genomic studies have provided a molecular basis for understanding the biosynthetic pathways of these volatile constituents; a chromosome-level genome assembly successfully identified candidate genes associated with terpene biosynthesis [18]. Consequently, any study claiming activity of *U. dermestoides* extracts should specify whether the preparation process retained, removed, or transformed the volatile defensive components [32,33].

### 2.2. Phenolic Constituents and Other Small Molecules

Methanol and hexane extracts contain both overlapping and distinct small molecules. Gas chromatography-mass spectrometry (GC-MS) analysis revealed that limonene, myristic acid, palmitic acid, stearic acid, oleic acid, and linoleic acid were detectable in both types of extracts; 1-pentadecanol, α-pinene, β-phellandrene, and α-terpinene were predominantly found in the methanol extract; whereas 2-methyl-1,4-benzoquinone, 2,4-dihydroxy-1-ethylbenzene, and dimethylquinone-related compounds were detected in the hexane extract [12,34]. At this stage, these data should be interpreted primarily as compositional evidence rather than proof that any single molecule accounts for the activity of crude extracts. In the hen’s egg test on the chorioallantoic membrane (HET-CAM) assay, the methanol extract displayed potential anti-irritant activity, whereas the hexane extract did not; however, phenolic fractions derived from acetone and ethanol crude extracts were observed to exhibit cytotoxicity and genotoxicity in HaCaT keratinocytes [35]. This suggests that phenolic fractions rich in antioxidant components may exert pro-oxidant or genotoxic effects depending on concentration, cellular context, and metabolic activation conditions [36].

Furthermore, the resource availability of flavonoids from *U. dermestoides* has also attracted attention. A study employed single-factor experiments and response surface methodology to investigate the extraction process of *U. dermestoides* flavonoids and determined that the optimal extraction conditions were 52.6% ethanol as the extraction solvent, a temperature of 82 °C, a solid-to-liquid ratio of 1:15, and an extraction time of 2 h [37]. This indicates that *U. dermestoides* contains not only volatile quinones and terpenes but also holds potential for the development of flavonoid-based antioxidant substances.

### 2.3. Lipid Fraction and Fatty Acids

Jasso-Villagomez et al. obtained the lipid fraction of *U. dermestoides* by Soxhlet extraction, with a total lipid content of 24.7 ± 0.7% [12]. GC-MS analysis revealed that this lipid fraction contained 31 fatty acids, with the major constituents being a mixture of linoleic acid and oleic acid (accounting for 40.9%), palmitic acid (31.9%), and stearic acid (9.3%); additionally, undecane, dodecanoic acid, tetradecanoic acid, heptadecanoic acid, eicosenoic acid, docosanoic acid, various long-chain alkanes, and trace steroidal substances were detected [12]. Despite its body length of only approximately 0.6 cm, *U. dermestoides* contains abundant unsaturated fatty acids, and its potential applications in skincare, hyperlipidemia, and malignant tumors have attracted attention [22].

### 2.4. Proteins, Bioactive Peptides, and Antioxidant Enzyme Complexes

Proteins and peptides represent a highly promising direction in the development of *Ulomoides dermestoides*. In the fields of functional foods and nutritional chemistry, the protein extracts and hydrolysates of *U. dermestoides* have also garnered considerable interest. In systematic studies of insect natural product chemistry, *U. dermestoides* has been recognized as a potential insect pharmacological resource with antioxidant and antimicrobial activities [38]. Recent studies have indicated that *U. dermestoides* contains antimicrobial peptides, lectins, and immune proteins [38,39].

Flores et al. extracted proteins from adult *U. dermestoides* using phosphate-buffered saline (PBS) buffer, and sodium dodecyl sulfate-polyacrylamide gel electrophoresis (SDS-PAGE) revealed strong protein bands at 130 kDa and 45 kDa [40]. Hydrolysis of the protein extract with Aspergillus oryzae protease released peptides exhibiting significant antioxidant activity. Chemical analysis showed that the 2,2′-azino-bis(3-ethylbenzothiazoline-6-sulfonic acid) (ABTS) radical scavenging activity of the *U. dermestoides* protein extract was 54.8 ± 3.1 μmol Trolox/g, which was markedly enhanced to 104 ± 6.2 μmol Trolox/g after enzymatic hydrolysis. The study also found that flavonoids and tannins, which are commonly associated with antioxidant activity, were not detected in the insect extract, indicating that the observed antioxidant activity was primarily attributable to the protein and peptide fractions. Additionally, the *U. dermestoides* protein hydrolysate exhibited antimicrobial activity against microorganisms such as *Proteus vulgaris*, *Shigella flexnerii*, and *Bacillus* spp. [40,41,42].

Ushakova et al. prepared an aqueous extract of *U. dermestoides* using an electro-pulse plasma dynamic extraction method and found that it contained antioxidant enzyme complexes [14]. Proteomic analysis revealed that this aqueous extract contained a variety of antioxidant and stress-related proteins, including SOD, catalase, peroxiredoxin, glutathione S-transferase, and heat shock proteins (HSP60, HSP70), along with non-protein antioxidants such as ethylhydroquinone [14,43]. Chemiluminescence assays demonstrated that the antioxidant activity of the aqueous extract at 1 mg/mL was equivalent to 0.2 mM Trolox [14]. In the *Caenorhabditis elegans* model, this extract prolonged the median lifespan by 34% under normal conditions and by 17% under oxidative stress induced by 50 mM paraquat [14,44]. Subsequent studies further purified antioxidant protein complexes from larval aqueous extracts, confirming their synergistic antioxidant effects [43]. The genetic basis for the presence and expression of these antioxidant protein complexes is explored in the following subsection (Section 2.7), which examines the genomic landscape of detoxification and antioxidant gene families in *U. dermestoides*.

### 2.5. Glycoproteins and UGP2A

Macromolecular glycoconjugates are closely associated with the anti-inflammatory and vascular protective effects of *Ulomoides dermestoides*. Yu et al. isolated and structurally characterized a glycoprotein designated UGP2A from *U. dermestoides* through aqueous extraction, ethanol precipitation, and chromatographic purification [21]. Characterization by High-performance gel permeation chromatography (HPGPC), ultraviolet (UV), circular dichroism, Fourier-transform infrared spectroscopy (FT-IR), Scanning electron microscopy (SEM), and Nuclear magnetic resonance (NMR) revealed that UGP2A is a protein-dominant glycoprotein containing sugar and protein moieties, O-glycosidic linkages, and a triple helix-related conformation [21]. This glycoprotein attenuated lipopolysaccharide (LPS)-induced acute lung injury, suppressed pulmonary edema and inflammatory cell infiltration, and reduced the levels of pro-inflammatory cytokines, including tumor necrosis factor-α (TNF-α), interleukin-1β (IL-1β), and interleukin-6 (IL-6). In LPS-stimulated RAW264.7 macrophages, UGP2A inhibited Inducible nitric oxide synthase (iNOS) and Cyclooxygenase-2 (COX-2) expression and blocked Nuclear factor kappa-B (NF-κB) p65 nuclear translocation. Further mechanistic studies indicated that UGP2A may inhibit endothelial activation and inflammation through the TLR4/MyD88 signaling pathway [21,45,46].

### 2.6. Integrated Summary of Chemical Classes, Pharmacological Activities, and Translational Considerations

#### Dual Nature of Bioactive Compounds: Antioxidant Versus Cytotoxic/Genotoxic Effects

A critical issue emerging from the literature is the apparent duality of certain bioactive compounds—particularly benzoquinones and phenolic constituents—which exhibit both antioxidant and cytotoxic/genotoxic activities depending on concentration, cellular context, and experimental conditions. Benzoquinones (MBQ and EBQ) reportedly exhibit antioxidant activity at low concentrations through ROS scavenging, yet induce cytotoxicity and DNA damage at higher concentrations in A549 cells (IC_50_ = 0.26 equivalent/mL) and normal monocytes [10]. Similarly, phenolic fractions show anti-irritant activity in the HET-CAM assay but concentration-dependent cytotoxicity and genotoxicity in HaCaT keratinocytes. This concentration-dependent duality is consistent with the broader toxicological literature on quinones, which act through redox cycling: at low concentrations, they stimulate adaptive stress responses (hormesis); at higher concentrations, they overwhelm cellular defenses, leading to oxidative damage and apoptosis. This paradox underscores that crude extract bioactivity likely represents a net effect of competing pro- and antioxidant activities, that the therapeutic window is likely narrow, and that quantitative limits for benzoquinone content in finished products are essential for safety. Furthermore, the reported effects were observed in different experimental systems—cytotoxicity in cancer cell lines and monocytes versus antioxidant effects in cell-free assays, *C. elegans*, and rodent models—complicating direct comparisons due to differences in bioavailability, metabolism, and cellular uptake. Future studies should systematically investigate concentration–response relationships for individual compounds across multiple cell types to define the therapeutic window and selectivity index, and elucidate the molecular mechanisms underlying this duality. Table 1 provides an integrated summary of the chemical classes, pharmacological activities, and translational considerations for each compound class discussed in this review.

**Table 1:** Representative bioactive substance classes, characteristics, and translational bottlenecks of *Ulomoides dermestoides*.

Class	Subclass	Representative Molecule/Marker	Evidence Type *	Extraction/Detection Method	Main Pharmacological Activity	Safety/Toxicity	Key Translational Bottleneck	References
Volatile quinones, alkenes, and terpenes	Benzoquinones	MBQ, EBQ, p-benzoquinone	I (Isolated)	Dichloromethane extraction; HS-SPME; GC-MS	Defensive volatiles; antimicrobial; repellent	Cytotoxicity, DNA damage (A549 cells)	Genotoxic risk; volatile or transformed during processing	[10]
	Alkenes	1-Pentadecene, 1-tridecene	II (Extract)	HS-SPME; GC-MS	Repellent, pheromone function	Low toxicity (no cytotoxicity)	Single function; synergistic effects require verification	[8]
	Terpenes and derivatives	Limonene, α-pinene, α-terpinene, carvone, geranial, geraniol, etc.	II (Extract)	Steam distillation; HS-SPME; GC-MS	Anti-inflammatory, antibacterial, antioxidant	Some terpenes (e.g., limonene) GRAS-certified; possible irritation at high concentrations	Distinguishing single-component vs. synergistic activities is still needed	[8,16]
	Alkyl disulfides	Diheptyl disulfide, etc.	-	Essential oil extraction; GC-MS	Antibacterial, repellent	–	Functional validation insufficient	[8]
Fatty acids and lipids	Saturated fatty acids	Palmitic acid, stearic acid, myristic acid	II (Extract)	Soxhlet extraction (petroleum ether); GC-MS	Energy substrates; membrane structural components	Risk of pro-inflammation with high saturated fatty acid intake	Poor oxidative stability; activity dependent on complex matrix	[12]
	Unsaturated fatty acids	Oleic acid, linoleic acid	II (Extract)	Same as above	PPARγ agonism; hypoglycemic, improved insulin sensitivity	Well tolerated within safe doses	Comparison of purified fatty acids vs. total lipid activity missing	[12]
Proteins and enzymes	Antioxidant enzymes	SOD, catalase, peroxiredoxin, glutathione S-transferase	II (Extract)	EPDE aqueous extraction; LC-MS/MS proteomics	Enzymatic ROS scavenging; antioxidant activity (Trolox equivalent ~2.1 mM)	No direct cytotoxicity	Low oral bioavailability; in vivo targets unclear	[14,43]
	Stress proteins	HSP60, HSP70, HSP83	III (Proteomic)	Same as above	Cytoprotection, stress resistance	–	Functional validation lacking	[43]
	Other functional proteins	Calmodulin, cytochrome c-2, nucleoside diphosphate kinase	-	Same as above	Cell signal transduction; energy metabolism	–	Pharmacological relevance unclear	[43]
Enzymatic hydrolysate peptides	Low-molecular-weight peptides	<15 kDa peptides	II (Extract)	PBS extraction + *Aspergillus oryzae* hydrolysis; Tris-Tricine SDS-PAGE	Antioxidant (ABTS, DPPH), antibacterial (Proteus, Shigella, Bacillus)	No obvious toxicity reported	Peptide sequences unknown; digestive stability and absorption mechanisms to be investigated	[40]
Glycoprotein	–	UGP2A (O-glycosidic linkage, predominantly β-sheet, 38.34% carbohydrate + 57.90% protein)	I (Isolated)	Aqueous extraction–ethanol precipitation–DEAE-ff and Sephadex G-100 column chromatography; HPGPC/UV/(circular dichroism) CD/FT-IR/SEM/NMR	Anti-inflammatory (inhibits IL-1β-induced upregulation of vWF, P-selectin, IL-6, NF-κB); antithrombotic (Deep vein thrombosis (DVT) model); anti-platelet activation; TLR4/MyD88 pathway	Favorable in vitro/in vivo safety (no cytotoxicity, no hemolysis, normal hepatic/renal function and histology)	Oral PK/PD data missing; long-term immunogenicity assessment needed	[21]
Phenolics and flavonoids	Phenolics	2-Methyl-1,4-benzoquinone, 2,4-dihydroxy-1-ethylbenzene, 2,5-dimethylquinone	II (Extract)	Methanol/hexane extraction; GC-MS	Anti-irritant (HET-CAM, methanol extract IR = 3.09 ± 0.11, comparable to nimesulide)	Some phenolics show cytotoxicity and genotoxicity (HaCaT cells)	Pro-oxidant risk; topical safety requires evaluation	[47]
	Flavonoids	Total flavonoids (monomers to be identified)	III (Chemical process)	Ethanol reflux extraction; response surface optimization	Antioxidant (DPPH assay)	Not applicable	Incompletely characterized constituents; in vivo activity unconfirmed	[48]
Genomic and omics resources	–	15,553 protein-coding genes; 11 chromosomes (incl. XY); genome size of 253.38 Mb; repetitive sequences account for 48.11%	III (Genomic)	Illumina short-read + PacBio HiFi + Hi-C sequencing	Provides molecular basis for biosynthetic pathway studies of bioactive substances (e.g., candidate genes for terpene synthesis)	Not applicable	Functional genome annotation requires further validation; gene presence does not confirm function	[18]

* Evidence Type Key: Level I = Isolated/purified compound with direct experimental evidence; Level II = Extract-level evidence with reproducible activity; Level III = Correlative/hypothesis-generating evidence (genomic, computational, homology-based). This table integrates chemical, pharmacological, and translational information across all compound classes discussed in this review.

### 2.7. Genomic Landscape of Detoxification and Antioxidant Gene Families

Building upon the proteomic identification of antioxidant proteins presented in Section 2.4, a more fundamental question arises: whether the high expression of these proteins has a genetic basis. The aqueous extract of *U. dermestoides* has been confirmed at the proteomic level to contain complexes of various antioxidant-related proteins, including superoxide dismutase (SOD), catalase (CAT), glutathione S-transferase (GST), and heat shock proteins (HSPs), and its ROS-scavenging activity has been well validated in both in vitro and in vivo models [14,43]. These findings raise a more fundamental question: whether the high expression of these antioxidant proteins has a genetic basis. The chromosome-level genome of *U. dermestoides* released in 2024 (BioProject PRJNA1135335) provides a critical resource for exploring this question [18]. This genome spans 253.38 Mb and contains 15,553 predicted protein-coding genes, with a BUSCO completeness score of 99.62%, indicating a high-quality assembly [18]. However, it is important to emphasize that the presence of homologous genes does not, by itself, constitute proof of biological function or pharmacological relevance. A complete evidentiary chain—from gene transcription to protein translation, post-translational modification, enzymatic activity, bioavailability, and target tissue effect—remains to be established.

Notably, homologs of antioxidant enzyme genes, including SOD, CAT, GST, HSP, and peroxiredoxin (PRX), have been identified in the *U. dermestoides* genome. To facilitate the interpretation of these genes, we drew upon the genomic data of *Tribolium castaneum*, a closely related species within the same family Tenebrionidae, which serves as the most well-characterized genomic reference for this taxonomic group. In the genome of T. castaneum, multiple SOD transcripts exist, including extracellular Cu/Zn-SOD, intracellular Cu/Zn-SOD, and mitochondrially localized Mn-SOD, with up to three transcript variants for extracellular SOD [49]; moreover, 36 cytosolic and 5 microsomal GST genes have been identified [50]. The CAT gene is stably present as a single copy in multiple tenebrionid species and is upregulated under oxidative stress conditions [51]. Given the conservation of gene families within Tenebrionidae, the *U. dermestoides* genome likewise encodes homologous genes for the above functional proteins. These genomic findings should be interpreted as a potential genetic basis for observed antioxidant activities, rather than as direct mechanistic evidence. The correlation between gene presence and proteomic identification of corresponding proteins in the aqueous extract supports the plausibility of this interpretation, but definitive conclusions regarding causality require functional validation through gene expression analysis (qPCR), enzymatic activity assays, and, ideally, knockdown or knockout experiments.

In insects, SOD and CAT constitute a two-tier enzymatic defense system for scavenging reactive oxygen species: SOD dismutates superoxide anions into H_2_O_2_, and CAT further decomposes H_2_O_2_ into water and oxygen. Their synergistic action can significantly reduce intracellular peroxide levels. The substrate diversity of *U. dermestoides* GST genes and their role in oxidative stress protection have been supported by prior research. Notably, in T. castaneum, the key gene Laccase2 has been identified to be involved in the biosynthesis of benzoquinone-defensive substances; this enzyme is expressed in the terminal secretory cells of odoriferous glands and is responsible for safely processing harmless precursors into toxic *p*-benzoquinone [52]. Elucidating the genetic basis for the safe production of toxic compounds provides important insights into the biosynthetic and transport mechanisms of *U. dermestoides* defensive secretions (benzoquinones).

HSP70 and HSP60 are among the most abundant stress proteins in the aqueous extract of *U. dermestoides* [14,43]. Recent reviews have clearly indicated that HSPs can indirectly modulate ROS levels by regulating the NF-κB signaling pathway, and HSP60 plays a protective role in mitochondrial oxidative phosphorylation [51]. The co-expression of HSP60 and HSP70 with antioxidant enzymes such as SOD and CAT in the aqueous extract suggests a potential functional synergy: SOD and CAT directly scavenge ROS generated during redox reactions, while HSPs, by maintaining protein homeostasis and modulating stress signaling pathways, suppress secondary oxidative damage induced by protein misfolding and mitochondrial dysfunction.

**Figure 2:**
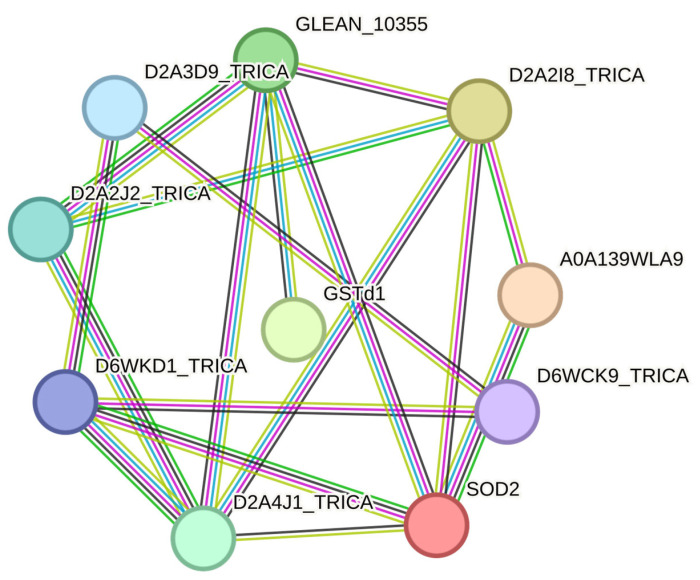
STRING-predicted protein–protein interaction network of antioxidant-related proteins identified in *Ulomoides dermestoides* aqueous extract (based on *Tribolium castaneum* homologs). Nodes represent individual proteins (labeled with *T. castaneum* identifiers), edges indicate predicted functional associations (confidence ≥ 0.7). The network reveals close interconnections among ROS-scavenging enzymes (SOD, CAT), phase II detoxification enzymes (GST), and stress chaperones (HSP70), supporting the multi-component synergistic antioxidant and anti-inflammatory mechanisms of *U. dermestoides*.

Figure 2 presents the predicted protein–protein interaction network of antioxidant proteins in the aqueous extract of *U. dermestoides* based on the STRING database. SOD, CAT, GST, and HSP70 exhibit high interconnectivity, supporting the hypothesis of a SOD–CAT–HSP synergistic mechanism for ROS scavenging and inflammation regulation.

**Figure 3:**
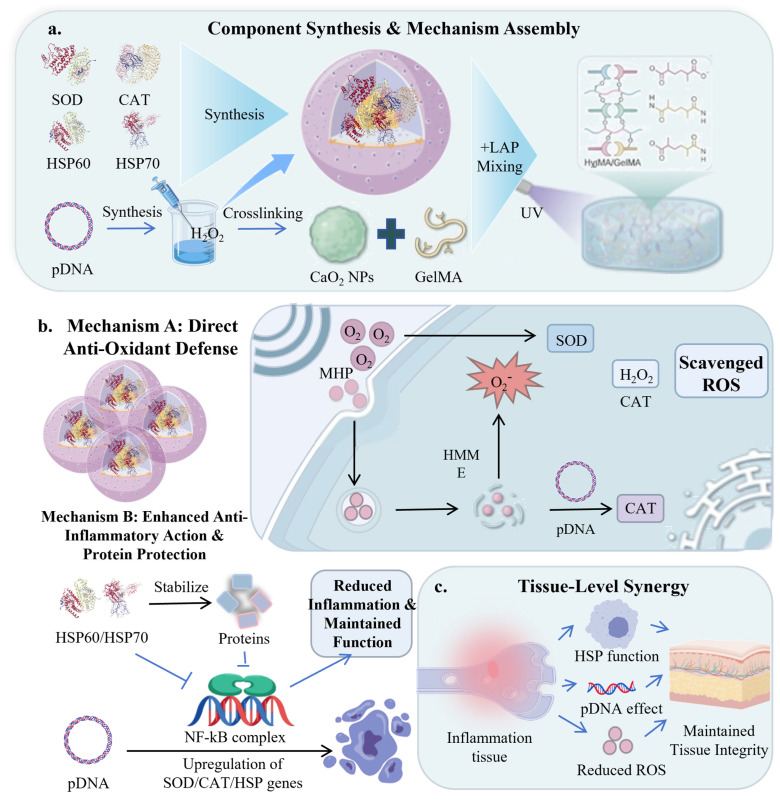
Schematic diagram of the synergistic antioxidant and anti-inflammatory mechanisms of SOD−CAT−HSP in *Ulomoides dermestoides*. The figure is organized into three panels (**a**–**c**) representing component integration, direct antioxidant defense, and tissue-level synergy, respectively. Under oxidative stress (**left panel**), superoxide dismutase (SOD) dismutates superoxide anion (O_2_^−^) to hydrogen peroxide (H_2_O_2_), which is further decomposed to water and oxygen by catalase (CAT), completing a two-phase direct ROS scavenging cascade. Concurrently, stress signals activate HSP60 and HSP70; HSP70 inhibits NF−κB nuclear translocation, thereby attenuating inflammatory responses. Genomic and proteomic evidence supports functional synergy among multiple protein families (SOD, CAT, GST, HSP), forming a multi-layer defense network that spans from gene encoding to protein expression, and from enzymatic ROS clearance to inflammation regulation. The model integrates genomic/proteomic observations and requires further validation at the protein-interaction and in vivo target-tissue levels.

Figure 3 summarizes the synergistic mechanism of the three protein families SOD, CAT, and HSP in the aqueous extract of *U. dermestoides*. Under oxidative stress, SOD dismutates superoxide anions into hydrogen peroxide, which is subsequently decomposed by CAT into water and oxygen, completing two-tier ROS scavenging; concurrently, HSP70 alleviates inflammatory responses by inhibiting NF-κB nuclear translocation, while HSP60 maintains mitochondrial function.

In summary, the encoding of genes related to SOD, CAT, GST, PRX, HSP, and others in the *U. dermestoides* genome provides a reliable genetic explanation for the antioxidant activities detected at the protein level in the aqueous extract. However, several critical questions remain unanswered: Are these genes actively transcribed and translated under relevant conditions? What are the post-translational modifications and enzyme kinetics of the expressed proteins? What is the oral bioavailability of these protein components when administered as crude extracts? Addressing these questions represents a priority for future research. The genomic data should be viewed as a valuable resource for hypothesis generation and as a foundation for targeted functional studies, rather than as definitive mechanistic proof. The direct ROS scavenging by SOD and CAT, the synergistic detoxification by GST, and the stress protection by HSPs together constitute a multi-layered antioxidant defense network spanning from gene coding to protein expression, and from enzymatic clearance to stress protection. Incorporating these key antioxidant enzyme genes into the quality marker screening system for *U. dermestoides* bioactive substances may provide precise molecular targets for the development of standardized functional products.

### 2.8. Molecular Docking Methodology and Interpretation

To complement genomic and proteomic evidence, we performed molecular docking predictions to explore potential molecular-level interactions between *U. dermestoides*-derived compounds and key antioxidant/anti-inflammatory targets. Docking was conducted using CB-Dock2 (https://cadd.labshare.cn/cb-dock2/index.php, (accessed on 25 April 2026)), an automated docking server that employs AutoDockTools-1.5.7 for docking calculations and CurPocket for cavity detection.

Ligand Preparation: Three-dimensional structures of representative compounds (MBQ, EBQ, linoleic acid, oleic acid, stearic acid, azelaic acid, m-cresol, 2,4-dihydroxyethylbenzene) were obtained from PubChem and optimized using the MMFF94 force field in Chem3D. Ligands were prepared in SDF format with energy minimization. Protein Structure Sources: Target protein structures were retrieved from the Protein Data Bank (PDB) with the following IDs: SOD (PDB: 3QFP), TLR4/MD-2 (PDB: 3FXI), NF-κB p50 (PDB: 1SVC), PPARγ (PDB: 4EMA), and Kelch-like ECH-associated protein 1 (Keap1) (PDB: 2FLU). For proteins with multiple available structures, the highest-resolution crystal structures were selected. Docking Parameters: The docking box was defined to cover the known binding sites or active sites of each target protein, with grid spacing set to 1.0 Å. AutoDock Vina was used with exhaustiveness = 8 and number of binding modes = 10. The CB-Dock2 server reports binding affinity as ΔG (kcal/mol) and provides cavity sizes (Å^3^) and the top nine binding modes.

Binding Energy Thresholds: As a hypothesis-generating tool, binding affinities were interpreted as follows: ΔG < −6.0 kcal/mol indicated moderate binding potential; ΔG < −8.0 kcal/mol indicated strong binding potential. These thresholds are based on standard practices in computational drug discovery but should not be interpreted as evidence of actual biological activity. Validation and Controls: No experimental validation (e.g., surface plasmon resonance (SPR), isothermal titration calorimetry (ITC)) was performed. The docking results should be considered as computational predictions requiring experimental confirmation, and are presented solely to generate testable hypotheses for future mechanistic studies. We explicitly acknowledge that molecular docking does not account for solvation effects, protein flexibility, or cellular context, and that predicted binding does not necessarily translate to functional modulation in biological systems.

**Figure 4:**
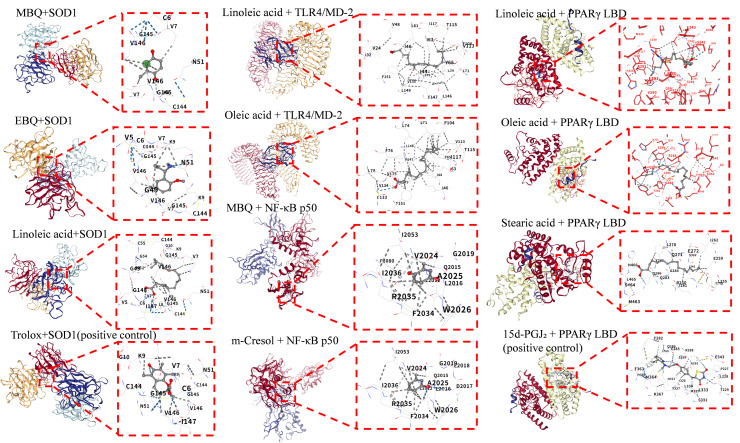
Molecular docking predictions of *Ulomoides dermestoides*-derived compounds with key antioxidant and anti-inflammatory targets. Hydrogen bonds and hydrophobic interactions are shown as dashed lines. These computational predictions are hypothesis-generating only and provide molecular-level hypotheses for the synergistic antioxidant (SOD, Keap1), anti-inflammatory (TLR4, NF-κB), and metabolic (PPARγ) activities of *U. dermestoides* extracts. The results require experimental validation through techniques such as surface plasmon resonance (SPR), isothermal titration calorimetry (ITC), or enzyme activity assays before any mechanistic conclusions can be drawn.

Figure 4 summarizes the binding modes of *U. dermestoides*-derived compounds (MBQ, EBQ, linoleic acid, oleic acid, stearic acid, azelaic acid, *m*-cresol, 2,4-dihydroxyethylbenzene, etc.) with key antioxidant/anti-inflammatory targets such as SOD, TLR4/MD-2, NF-κB p50, PPARγ, and Keap1, providing molecular-level complement to the preceding genomic and proteomic evidence and echoing the potential mechanism of multi-component synergistic effects.

## 3. Molecular Mechanisms of Antioxidant and Anti-Inflammatory Activities

**Figure 5:**
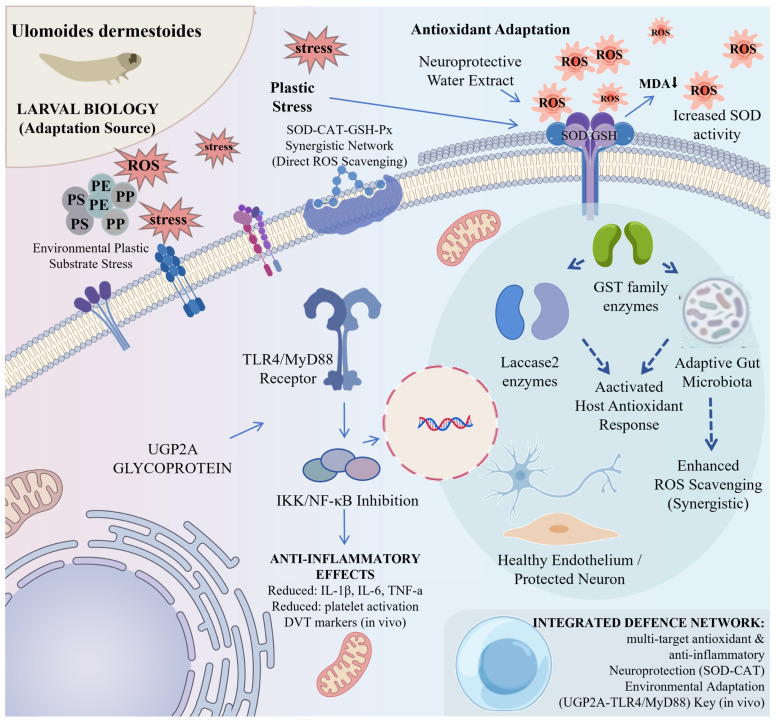
Schematic diagram of the multi-layer molecular mechanisms underlying antioxidant and anti-inflammatory activities of *Ulomoides dermestoides*. Under plastic substrate (polystyrene (PS), polyethylene (PE), polypropylene (PP)) stress, larvae activate the SOD-CAT-GSH-Px synergistic network for direct ROS scavenging and malondialdehyde (MDA) reduction, while upregulating GST family enzymes and Laccase2. The O-glycosylated UGP2A glycoprotein activates the host antioxidant response via TLR4; enhances ROS scavenging; inhibits IKK/NF-κB signaling; reduces IL-1β, IL-6, and TNF-α; protects endothelium and neurons; and suppresses platelet activation and DVT markers. The bottom part integrates the two core axes—neuroprotection (SOD-CAT) and environmental adaptation (UGP2A-TLR4/MyD88)—forming a multi-target integrated defense network for antioxidant and anti-inflammatory effects. Solid lines represent experimentally validated pathways (supported by in vitro, in vivo, or proteomic evidence), whereas dashed lines represent hypothesis-generating connections derived from omics or computational analyses that require experimental validation.

This chapter focuses on the synergistic mechanisms of these bioactive components at the cellular and molecular levels. By integrating recent evidence on neuroprotection, glycoprotein signaling pathways, environmental stress responses, and gut microbiota interactions, we aim to construct a unified multi-target molecular model of the antioxidant and anti-inflammatory effects of *Ulomoides dermestoides*.The multi-layer molecular mechanisms integrating direct ROS scavenging, anti-inflammatory signaling, and environmental adaptation are summarized in Figure 5.

### 3.1. Direct ROS Scavenging: The SOD–CAT Synergistic Network

The aqueous extract of *Ulomoides dermestoides* exhibits significant neuroprotective effects in a paraquat-induced Parkinson’s disease-like model. Ambaryan et al. reported that the larval aqueous extract of *U. dermestoides* (0.4 mg/kg) significantly improved motor coordination and object recognition performance in model mice, and this effect was closely associated with reduced oxidative stress in the nigrostriatal region [44]. Mechanistically, the aqueous extract increased antioxidant enzyme activities and decreased malondialdehyde (MDA) content in the brain tissue of model animals, suggesting that the SOD–CAT synergistic antioxidant network (see Section 2.4) plays a central role in neuroprotection [43,44,53].

Notably, the antioxidant defense system of *U. dermestoides* is not merely present in extracts but can be significantly activated under environmental stress. Multiple studies have demonstrated that *U. dermestoides* larvae can effectively degrade plastic polymers such as polystyrene (PS), polyethylene (PE), and polypropylene (PP) [54]. During plastic degradation, oxidoreductases (e.g., laccase, peroxidase) initiate oxidative cleavage of polymer C–C bonds, a process accompanied by substantial generation of reactive oxygen species (ROS). Gut microbiome analysis revealed that plastic substrates markedly altered the gut microbial community composition of *U. dermestoides* larvae, with pronounced changes in the abundance of bacterial taxa associated with oxidative stress responses [55]. These findings indicate that antioxidant enzyme systems such as SOD and CAT in *U. dermestoides* not only participate in maintaining basal redox homeostasis but can also be induced under xenobiotic stress, thereby protecting cells from ROS-induced damage. From an ecological adaptation perspective, this highly efficient antioxidant defense capability may represent a key adaptive trait that enables *U. dermestoides* to survive in diverse extreme environments.

### 3.2. UGP2A Glycoprotein-Mediated TLR4/MyD88 Anti-Inflammatory Signaling Pathway

In 2026, Yu et al. isolated and characterized a novel glycoprotein, UGP2A, from *U. dermestoides*, providing the clearest molecular pathway evidence to date for its anti-inflammatory mechanisms [21]. In that study, UGP2A was obtained through aqueous extraction, ethanol precipitation, and chromatographic purification, and was confirmed by HPGPC, UV, circular dichroism (CD), FT-IR, SEM, and NMR as a protein-dominant glycoprotein with O-glycosidic linkages (containing 38.34% carbohydrate and 57.90% protein).

In a mouse model of deep vein thrombosis (DVT), UGP2A significantly inhibited thrombus formation, reduced inflammatory markers, and suppressed platelet activation. In IL-1β-stimulated human umbilical vein endothelial cells (HUVECs), UGP2A inhibited the upregulation of von Willebrand factor (vWF), P-selectin, IL-6, and TNF-α, and blocked the nuclear translocation of NF-κB-related signals. The authors further demonstrated that the anti-inflammatory effects of UGP2A are partially dependent on the TLR4/MyD88 signaling pathway. Safety evaluation, encompassing cytotoxicity, hemolysis, hepatic and renal function, histopathology of major organs, and tail bleeding assays, confirmed that UGP2A possesses favorable biosafety both in vitro and in vivo [21,56,57]. This finding directly links *U. dermestoides* to innate immune signaling and thromboinflammatory endothelial dysfunction, providing a mechanistic explanation for its traditional use in promoting blood circulation and resolving stasis.

### 3.3. Coupling of Antioxidant and Detoxification Systems

The potent antioxidant capacity displayed by *U. dermestoides* during plastic degradation is intimately linked to the detoxification enzyme gene families encoded in its genome. The chromosome-level genome assembly (253.38 Mb, 15,553 protein-coding genes) has identified multiple gene families associated with oxidative stress and xenobiotic metabolism [18]. Among them, genes such as Laccase2 are responsible for converting harmless precursors into toxic benzoquinone-defensive substances, whereas members of the GST family participate in the detoxification of reactive intermediates generated during metabolism [18,58]. In closely related species such as *Tribolium castaneum*, 36 cytosolic and 5 microsomal GST genes have been identified, and homologous genes are also present in the *U. dermestoides* genome. This closed-loop system enables *U. dermestoides* to produce chemical defensive substances while averting autotoxicity.

Furthermore, the gut microbiome plays an important role in the antioxidant defense of *U. dermestoides*. Upon exposure of *U. dermestoides* larvae to plastic films such as thermoplastic cassava starch or polylactic acid composite films, the diversity and composition of their gut microbial communities underwent significant changes, with an increased relative abundance of certain bacterial taxa associated with antioxidant enzyme production [55,59]. This finding suggests the possible existence of a synergistic antioxidant mechanism between the host and gut microbiota, jointly coping with environmentally induced oxidative stress.

### 3.4. Multiple Synergistic Mechanisms

Taken together, the antioxidant and anti-inflammatory activities of *U. dermestoides* involve multi-level synergies, which have been experimentally validated in multiple scenarios including neuroprotection, vascular protection, and environmental adaptation: enzymatic ROS scavenging via the SOD-CAT synergistic network (see Section 2.4 and Section 3.1) [43,44,54]; anti-inflammatory signaling through the UGP2A-TLR4/MyD88-NF-κB pathway [21]; metabolic regulation via the PPARγ/GLUT4 pathway [18]; and gut microbial synergy contributing to host antioxidant defense [55].

Notably, the antioxidant capacity of *U. dermestoides* may share a common genetic and biochemical basis with its ecological adaptability, such as plastic degradation. Insects capable of surviving in extreme environments generally possess greater antioxidant system reserves and inducibility than typical species. Therefore, plastic degradation studies provide important ecological evidence for the genetic basis of the antioxidant capacity of *U. dermestoides*. This feature provides a broader theoretical foundation for its application in the development of antioxidant functional products.The key experimental evidence supporting these molecular mechanisms is summarized in Table 2.

**Table 2:** Key experimental evidence for the antioxidant and anti-inflammatory molecular mechanisms of *Ulomoides dermestoides*.

Mechanism Category	Key Bioactive Component/Model	Molecular Target/Signaling Pathway	Main Biological Effect	Experimental Model/Evidence Type	References
Antioxidant/Neuroprotection	Larval aqueous extract	SOD, CAT, GSH-Px	Increased SOD/GSH-Px activities and decreased MDA content in brain tissue; improved motor coordination and cognitive function	Paraquat-induced Parkinson’s disease-like mouse model	[43,44]
Anti-inflammatory/Vascular protection	UGP2A (glycoprotein)	TLR4/MyD88, NF-κB	Inhibited upregulation of vWF, P-selectin, IL-6, and TNF-α; blocked NF-κB nuclear translocation; antithrombotic effect	DVT mouse model + IL-1β-stimulated HUVECs	[21]
Environmental adaptation/Detoxification	Larvae (whole insect) (plastic degradation)	Laccase, peroxidase, GST	Degraded PS, PE, and PP plastics; induced antioxidant enzyme systems; adaptive changes in gut microbiota	Plastic degradation experiments + 16S rRNA gut microbiome analysis	[54,55,60]
Genomic basis	Chromosome-level genome	SOD, CAT, GST, Laccase2, and other gene families	Annotated 15,553 protein-coding genes, providing a genetic basis for antioxidant defense and detoxification	Genome assembly and annotation	[18]

### 3.5. Evidence Hierarchy in Mechanistic Interpretations

A critical methodological consideration in interpreting the pharmacological activities of *U. dermestoides* is the distinction between evidence derived from crude extracts versus that from isolated/purified compounds. Throughout this review, we have adopted the following evidence classification:

Level I (Direct experimental evidence): Findings obtained using isolated, purified, or chemically synthesized compounds in well-controlled experimental systems with appropriate positive/negative controls.

Level II (Extract-level evidence): Pharmacological activities observed using standardized crude extracts (aqueous, lipid, protein fractions) where the active principle(s) have not been isolated but the preparation methodology is reproducible.

Level III (Inference/prediction): Mechanistic interpretations based on homology with related species, computational predictions (docking), or genomic presence of genes encoding proteins of interest, without direct experimental validation in *U. dermestoides*.

Where Level I evidence exists (e.g., UGP2A glycoprotein, MBQ/EBQ cytotoxicity), we have emphasized these findings. Where only Level II or Level III evidence is available (e.g., most antioxidant effects attributed to protein complexes, genomic inferences), we have explicitly noted the limitations and the need for validation.

In addition to these three experimental evidence levels, we introduce a fourth level (Level IV) specifically for human case reports and observational data. These represent a distinct category of evidence—they are neither controlled experiments nor computational predictions, but rather uncontrolled human observations that provide valuable safety signals while lacking the rigor of controlled clinical trials. This four-level framework is applied consistently in Table 5.

## 4. In Vivo Pharmacological Evidence and Disease Models with Supporting In Vitro Mechanistic Data

This chapter focuses primarily on the pharmacological effects and safety boundaries of *Ulomoides dermestoides* bioactive components in in vivo disease models, with emphasis on four pharmacological directions that have garnered relatively strong experimental evidence: neuroprotection, metabolic regulation and antidiabetic effects, vascular protection and antithrombotic activity, and cytotoxicity/genotoxicity alerts associated with benzoquinone and phenolic fractions. Methodologically, this chapter prioritizes in vivo data from animal experiments to distinguish them from the in vitro and computational predictions presented in Section 2 and Section 3. Where in vitro cell culture models (e.g., 3T3-L1 adipocytes, HUVECs) are discussed, they are included as supporting mechanistic evidence to complement and interpret the in vivo findings, rather than as primary evidence of pharmacological efficacy.

### 4.1. Neuroprotection: Parkinson’s Disease-like Model and Vascular Dementia

The neuroprotective effects of *U. dermestoides* have been validated in two independent neurological disease models: the paraquat-induced Parkinson’s disease-like model and the vascular dementia animal model. In the paraquat-induced Parkinson’s disease-like model, the larval aqueous extract of *U. dermestoides* (0.4 mg/kg) significantly improved motor coordination and object recognition performance in model mice [44]. Mechanistic studies revealed that this aqueous extract increased the activities of SOD and glutathione peroxidase (GSH-Px) and decreased MDA content in the brain tissue of model animals, indicating that its neuroprotective effect is closely associated with the attenuation of oxidative stress in the nigrostriatal region [43,44]. Notably, under oxidative stress induced by 50 mM paraquat, the same aqueous extract prolonged the median lifespan of *Caenorhabditis elegans* by 12–17% compared with the toxin-treated group alone [44,61], further supporting its antioxidant defense activity in a neurotoxicity model. Collectively, these findings demonstrate that the neuroprotective effects of the *U. dermestoides* aqueous extract are not attributable to a single antioxidant endpoint but rather represent the integrated outcome of synergistic actions among multiple antioxidant enzymes, including SOD, CAT, and GSH-Px.

In the vascular dementia model, *U. dermestoides* preparations also demonstrated significant cognitive protective effects. Pan et al. showed that *U. dermestoides* preparations reduced MDA and endothelin-1 levels, modulated the nitric oxide/nitric oxide synthase (NO/NOS) ratio, and exerted a certain histological protective effect on pyramidal neurons in the hippocampal CA1 region in a mouse model of chronic cerebral hypoperfusion [10]. Multiple studies using a D-galactose-induced natural aging model have also reported that *U. dermestoides* increased SOD and GSH-Px activities in serum and brain tissue, decreased lipid peroxidation levels, and modulated acetylcholinesterase activity [62,63,64]. Significant improvements in skin water content and hydroxyproline content were also observed in a skin aging model. The consistency of these findings across multiple aging-related endpoints provides valuable in vivo evidence for the potential application of *U. dermestoides* aqueous extract in neurodegenerative diseases.

The neuroprotective evidence from paraquat-induced Parkinson’s and vascular dementia models is promising, with consistent findings of improved behavioral outcomes and reduced oxidative stress markers. However, all data derive from crude aqueous extracts whose active principles remain unidentified; sample sizes were modest, blinding was not explicitly employed, and intraperitoneal administration bypasses oral bioavailability barriers. The dose–response relationship and optimal dosing remain unknown. Future studies should focus on bioassay-guided identification of active components, oral bioavailability assessment, and confirmation in larger, blinded studies across additional neurotoxicity models.

### 4.2. Metabolic Regulation and Antidiabetic Effects

The most robust metabolic evidence to date derives from a study by Jasso-Villagomez et al. using an alloxan-induced diabetic mouse model to investigate the lipid fraction. Alloxan is a selective pancreatic β-cell toxin that induces β-cell necrosis through ROS generation and serves as a classic model of type 1 diabetes. The researchers fractionated *U. dermestoides* into chitin, protein, and lipid fractions and administered them in acute and subacute regimens.

In the acute experiment, both the lipid fraction (16 mg/kg) and the protein fraction (16 mg/kg) significantly reduced blood glucose levels in diabetic mice. In the 30-day subacute experiment, daily oral administration of 16 mg/kg of the lipid fraction reduced blood glucose from 593 mg/dL to 120 mg/dL, restored serum insulin levels, and decreased water and food intake by 65.5% and 58.5%, respectively [12]. Hepatic histological analysis revealed that the lipid fraction partially restored the normal arrangement of hepatocyte cords and attenuated hepatocyte vacuolization; pancreatic histology showed that the number of islets increased from 11.5% to 41.8%, and the number of cells per islet also increased significantly, suggesting a potential islet-protective or regenerative effect of the lipid fraction [12].

To further elucidate the molecular mechanism underlying the in vivo antidiabetic effects observed in the mouse model, the lipid fraction was evaluated in the 3T3-L1 adipocyte model. In this in vitro mechanistic study, the lipid fraction (100 μg/mL) significantly upregulated the mRNA expression of PPARγ and GLUT4, reaching 175% and 166.6% of the control levels, respectively. In the 3T3-L1 adipocyte model, this lipid fraction (100 μg/mL) significantly upregulated the mRNA expression of PPARγ and GLUT4, reaching 175% and 166.6% of the control levels, respectively. GC-MS analysis showed that the major fatty acids in the lipid fraction were oleic acid and linoleic acid (together accounting for approximately 40.9%), palmitic acid (31.9%), and stearic acid (9.3%) [12,65]. These fatty acids are known to act as natural ligands for PPARγ; upon activation, PPARγ directly regulates the expression of genes involved in lipid metabolism and promotes GLUT4 translocation and expression, thereby enhancing glucose uptake in peripheral tissues.

It is important to distinguish between the in vivo effects observed in the alloxan model and the in vitro mechanistic data. The alloxan-induced diabetes model primarily reflects insulin deficiency resulting from β-cell toxicity and provides direct evidence for β-cell protective and insulin secretion-improving effects of the lipid fraction. In parallel, the PPARγ/GLUT4 upregulation observed in 3T3-L1 adipocytes—consistent with the known role of PPARγ as a master regulator of insulin sensitivity—suggests that the lipid fraction may also have potential insulin-sensitizing effects relevant to type 2 diabetes pathophysiology. However, since the alloxan model does not directly capture insulin resistance, the in vivo relevance of these PPARγ-mediated effects remains to be confirmed. Future studies should employ high-fat diet combined with low-dose streptozotocin-induced type 2 diabetes models and conduct insulin tolerance and glucose tolerance tests to comprehensively evaluate the impact of the *U. dermestoides* lipid fraction on insulin sensitivity.

The lipid fraction demonstrates consistent hypoglycemic effects in alloxan-induced diabetic mice, supported by PPARγ/GLUT4 upregulation in 3T3-L1 cells. However, the alloxan model reflects type 1 rather than type 2 diabetes pathophysiology; insulin tolerance and glucose tolerance tests were not performed. The lipid composition varied between batches, and the contribution of individual fatty acids remains unclear. Future work should employ high-fat diet/streptozotocin-induced type 2 diabetes models, conduct glucose/insulin tolerance tests, and perform dose–response studies to establish the therapeutic window.

### 4.3. Vascular Protection and Thromboinflammation (DVT Model, UGP2A)

The vascular protective effects of *U. dermestoides* can be understood at two levels: first, the traditional blood-activating effects reported in early studies; second, the modern molecular mechanistic elucidation represented by the UGP2A glycoprotein. Early Chinese-language studies reported that *U. dermestoides* prolonged coagulation time and bleeding time and reduced whole blood viscosity, plasma viscosity, fibrinogen concentration, and hematocrit in mice, suggesting that it may exert anticoagulant effects by modulating coagulation factors, platelet function, or erythrocyte rheological properties [66,67]. Although these studies predate current anticoagulation evaluation standards, they provide pharmacodynamic clues regarding the vasoactive properties of *U. dermestoides*.

In a mouse model of deep vein thrombosis (DVT), UGP2A significantly inhibited thrombus formation, reduced inflammatory markers, and suppressed platelet activation. In IL-1β-stimulated human umbilical vein endothelial cells (HUVECs), UGP2A inhibited the upregulation of von Willebrand factor (vWF), P-selectin, IL-6, and TNF-α, and blocked the nuclear translocation of NF-κB-related signals; its anti-inflammatory effects were partially dependent on the TLR4/MyD88 signaling pathway [21]. Safety evaluation confirmed that UGP2A exhibits favorable biosafety both in vitro and in vivo. This finding links the traditional blood-activating effects of *U. dermestoides* to the modern concept of thromboinflammation—the interplay among endothelial cells, leukocytes, platelets, and innate immune pathways—and provides a pathway-level molecular explanation for its vascular protective activity [68,69].

It should be noted that although the anticoagulant and anti-inflammatory activities of UGP2A are consistent across multiple experimental systems, bleeding risk represents a major safety concern for its clinical translation. Future studies should systematically evaluate the effects of UGP2A on prothrombin time, activated partial thromboplastin time, and platelet aggregation function, and its interactions with anticoagulant and antiplatelet drugs [70].

The vascular protective effects are supported by early blood coagulation studies and the recent mechanistic UGP2A study demonstrating antithrombotic activity via the TLR4/MyD88 pathway. However, early studies lack methodological detail, and UGP2A was administered intravenously, leaving oral bioavailability completely unknown. The DVT model represents a single thrombosis model; arterial thrombosis and bleeding risk remain unevaluated. Future studies should develop oral delivery systems for UGP2A, characterize oral pharmacokinetics, and conduct comprehensive bleeding risk assessment.

### 4.4. Cytotoxicity and Genotoxicity of Benzoquinone and Phenolic Fractions

Safety evaluation of *U. dermestoides* requires attention to two particular aspects: first, the toxicological evidence of bioactive components in the benzoquinone and phenolic fractions at the cellular and animal levels; second, the data on adverse reactions in humans through traditional dietary exposure. At the cellular and animal levels, Crespo et al. clearly demonstrated that the benzoquinone compounds (MBQ and EBQ) in the defensive secretion of *U. dermestoides* exhibited concentration-dependent cytotoxicity against A549 lung cancer cells (IC_50_ = 0.26 equivalent/mL) and induced significant DNA damage as detected by the comet assay [10]. A synthetic benzoquinone mixture produced similar effects, whereas 1-pentadecene alone was inactive, confirming that benzoquinones are the main source of cytotoxicity and genotoxicity. In normal monocytes, the defensive secretion at a low dose of 0.15 equivalent/mL also inhibited cell proliferation by 72.2 ± 2.7% and induced DNA damage [10]. Mendoza-Meza et al. also observed cytotoxicity and genotoxicity of phenolic fractions in HaCaT keratinocytes [71]. These findings indicate that crude preparations rich in benzoquinones and phenolics are unsuitable for direct use as oral pharmaceuticals. However, this does not negate the safety of other *U. dermestoides* fractions—such as the lipid fraction, protein hydrolysates, and the UGP2A glycoprotein—within appropriate dose ranges, as these have demonstrated good tolerability in the aforementioned experiments [21,71,72].

Regarding human adverse reaction data, the literature contains several noteworthy case reports. Martínez-Rodríguez et al. reported a 70-year-old male who ingested *U. dermestoides* larvae as an alternative therapy (3 larvae per day) for approximately one month; colonoscopy revealed nonspecific inflammation, congestion, and submucosal hemorrhage in the left colon, along with numerous undigested insect carcasses [73]. Natt et al. described a 66-year-old male who consumed *U. dermestoides* larvae daily for diabetes treatment and, after about one month, developed peripheral eosinophilia (white blood cell count of 24,810/μL with 28% eosinophils), bilateral ground-glass opacities on chest imaging, and 60% eosinophils in bronchoalveolar lavage fluid; symptoms resolved spontaneously after discontinuation of *U. dermestoides*, and a diagnosis of acute eosinophilic pneumonia was established [63]. Additionally, a case of palpable purpura following *U. dermestoides* ingestion has been reported [74]. From a public health perspective, an experimental study found that *U. dermestoides* can serve as an intermediate host for *Hymenolepis diminuta* under laboratory conditions; 92% of the insects developed cysticercoids, which developed into adult worms and produced eggs after inoculation into experimental rats [75]. This suggests that farmed or wild-caught *U. dermestoides* without strict quarantine may carry zoonotic parasites, posing an additional food safety risk.

Based on the above safety data, several cautious conclusions can be drawn: crude extracts or defensive secretions of *U. dermestoides* rich in benzoquinone and phenolic fractions exhibit clearly detectable genotoxicity and should not be used directly as raw materials for oral pharmaceuticals; isolated and purified fractions of *U. dermestoides*, such as the lipid fraction, protein hydrolysates, and the UGP2A glycoprotein, have shown good tolerability in existing animal studies and hold potential for further development; as a traditional edible insect, *U. dermestoides* carries definite risks of infection and allergy, especially under non-standardized rearing conditions; and any health products developed from *U. dermestoides* must be subject to a rigorous quality control system, including quantitative control of benzoquinone compounds, microbial and parasite testing, heavy metal screening, and allergenicity assessment. Historical records of traditional use alone cannot substitute for modern toxicological safety evaluation.

It is important to distinguish between case reports of adverse events and evidence of therapeutic efficacy. The human data currently available for *U. dermestoides* are limited to case reports of adverse reactions following ingestion. No controlled clinical trials have been published for any *U. dermestoides* preparation. Therefore, claims of therapeutic efficacy in humans cannot be made, and the available case reports should be interpreted as safety signals requiring further investigation rather than as evidence of clinical outcomes. The main in vitro and in vivo pharmacological findings and safety data are compiled in Table 3.

**Table 3:** Key experimental evidence for pharmacological activities and safety of *Ulomoides dermestoides*.

Pharmacological Category	Main Model	Route/Dose	Key Observational Endpoints	Main Effects	References
Neuroprotection	Paraquat-induced Parkinson’s disease-like mice	Larval aqueous extract, 0.4 mg/kg	Motor coordination, cognitive function	Behavioral improvement; ↑ brain SOD and GSH-Px activities; ↓ MDA	[43,44]
	Chronic cerebral hypoperfusion mice (vascular dementia)	Whole-insect powder, i.g.	Hippocampal CA1 neurons, MDA/ET-1	Histological protection; improved oxidative/endothelial markers	[15]
	D-Galactose-induced aging mice	Whole-insect powder	SOD, GSH-Px, MDA	↑ SOD and GSH-Px; ↓ MDA; modulation of AChE	[44,76]
Metabolic regulation/Antidiabetic	Alloxan-induced diabetic mice (acute)	Lipid/protein fraction, 16 mg/kg i.p.	Blood glucose (0–360 min)	↓ Blood glucose (active fractions: lipid/protein)	[12]
	Alloxan-induced diabetic mice (30-day)	Lipid fraction, 16 mg/kg/day p.o.	Blood glucose, insulin, hepatic/pancreatic histology	↓ Blood glucose by 80%; ↑ insulin; ↑ islet number	[12]
	3T3-L1 adipocytes	Lipid fraction, 100 μg/mL	PPARγ, GLUT4 mRNA	↑ PPARγ by 75%; ↑ GLUT4 by 66.6%	[12]
Vascular protection/Antithrombotic	DVT mouse model	UGP2A, i.v.	Thrombus formation, inflammatory markers	Antithrombotic; ↓ IL-6, TNF-α; ↓ vWF, P-selectin	[21]
	IL-1β-stimulated HUVECs	UGP2A	NF-κB nuclear translocation, adhesion molecules	↓ vWF, P-selectin, IL-6, TNF-α	[21]
Safety/Toxicity	A549 cells, monocytes	Benzoquinone-defensive secretion	Cell viability, DNA damage	IC_50_ = 0.26 equivalent/mL; ↑ DNA damage	[10,71]
	HaCaT cells	Phenolic fractions	Cell viability, genotoxicity	Concentration-dependent cytotoxicity and genotoxicity	[71]
	Human ingestion (case reports)	Larvae, 3 individuals/day for 1 month	Colonic mucosa, pulmonary imaging, skin	Nonspecific colitis; acute eosinophilic pneumonia; palpable purpura	[63,73,74]
	Experimental infection	Hymenolepis eggs, orally	Cysticercoid formation, adult development	92% formed cysticercoids; developed into adults and produced eggs	[75]

↑ indicates increase, ↓ indicates decrease.

### 4.5. Comprehensive Safety Assessment: Current Evidence and Critical Gaps

Safety evaluation of *U. dermestoides* requires systematic consideration across multiple toxicological domains. While certain purified fractions have shown favorable safety profiles, significant gaps remain, particularly regarding subchronic/chronic toxicity, genotoxicity, immunogenicity, and oral bioavailability. Table 4 presents a comprehensive summary of safety evidence, categorized by toxicological endpoint, and identifies priority areas for future investigation.

Based on the available evidence, several safety conclusions and recommendations can be made. Crude extracts or defensive secretions containing benzoquinone and phenolic fractions exhibit clearly detectable cytotoxicity and genotoxicity and should not be used directly as oral pharmaceutical raw materials without extensive processing to remove or inactivate these components. In contrast, purified fractions such as the lipid fraction, protein hydrolysates, and the UGP2A glycoprotein have demonstrated favorable safety profiles in existing animal studies and represent viable candidates for further development, provided that purity and batch-to-batch consistency are maintained. Rearing conditions significantly impact safety; standardized, controlled farming practices are essential to minimize contamination, reduce parasite risk, and ensure consistent product quality. The documented ability of *U. dermestoides* to serve as an intermediate host for H. diminuta [77] underscores the need for strict parasite screening. Allergy risk should not be underestimated; given the homology between tenebrionid and crustacean allergens, individuals with shellfish or dust mite allergies should avoid products containing *U. dermestoides* proteins unless allergenicity has been definitively excluded for the specific preparation. Priority studies for translational development include acute and subchronic oral toxicity studies (OECD 423/425 and 407) in rodents, Ames test and in vivo micronucleus assay for genotoxicity, allergenicity assessment (IgE binding, basophil activation), oral pharmacokinetic studies, reproductive toxicity (OECD 414/416), and establishment of acceptable daily intake (ADI) values. Table 5 classifies the evidence levels for each pharmacological activity based on the four-tier framework.

**Table 4:** Comprehensive safety assessment of *Ulomoides dermestoides*.

Toxicological Domain	Available Evidence	Evidence Quality	Key Findings	Critical Gaps	Priority for Future Studies
Acute toxicity	Limited	Low	No data available for whole insect or isolated fractions	No LD_50_ established	High
Subchronic toxicity	Indirect (related species)	Moderate	Z. atratus NOAEL = 5000 mg/kg/day (13 weeks) [77]	No direct data for *U. dermestoides*	High
Chronic toxicity	None	N/A	No studies available	Complete data gap	High
Genotoxicity	Available (benzoquinones/phenolics)	Moderate	MBQ/EBQ DNA damage in A549 and monocytes [10]; phenolic genotoxicity in HaCaT [71]	Pure compound testing; Ames test needed	High
Cytotoxicity	Available	Moderate	Benzoquinone IC_50_ = 0.26 equivalent/mL in A549 [10]	Selectivity index (cancer vs. normal cells)	Moderate
Immunogenicity	Indirect (*T. molitor*)	Moderate	Tropomyosin cross-reactivity with crustacean allergens [78]	Specific allergens in *U. dermestoides* not identified	High
Allergenicity	Indirect (*T. molitor*)	Moderate	Thermal processing (boiling) reduces but does not eliminate IgE binding [78]	Thermal/enzymatic processing effects on allergenicity	High
Hemocompatibility	Available (UGP2A)	High	UGP2A: no hemolysis, no tail bleeding prolongation [21]	Effects on PT, APTT, and platelet aggregation	Moderate
Hepatic/Renal toxicity	Available (UGP2A)	High	UGP2A: normal hepatic/renal function and histology [21]	Crude extract and other fractions not evaluated	Moderate
Reproductive toxicity	None	N/A	No studies available	Complete data gap	High
Oral bioavailability	None	N/A	No PK data available for any preparation	Complete data gap	Very High
Parasite risk	Available (experimental)	Moderate	92% cysticercoid development with H. diminuta under laboratory conditions [75]	Farming practices and parasite screening	High

**Table 5:** Evidence level classification for major pharmacological activities of *Ulomoides dermestoides*.

Pharmacological Activity	Evidence Level	Experimental Model/System	Key Findings	Confidence Rating *	Limitations
Antioxidant (enzymatic)	II (Extract)	In vitro chemiluminescence	1 mg/mL aqueous extract = 0.2 mM Trolox equivalent	High (reproducible)	Crude extract; synergistic components not identified
Antioxidant (enzymatic)	II (Extract)	C. elegans model	34% lifespan extension under normal condition; 17% under paraquat stress	High (well-validated model)	Mechanism in intact organism not fully elucidated
Antioxidant (enzyme complex)	II (Extract)	Proteomic identification	SOD, CAT, PRX, GST, HSP60/HSP70 in aqueous extract	Moderate (correlative)	Functional synergy not directly proven
Neuroprotection	II (Extract)	Paraquat-induced Parkinson’s mouse model	Improved motor/cognitive function; ↑ SOD/GSH-Px; ↓ MDA	High (in vivo effects)	Crude extract; active principle unknown
Neuroprotection	II (Extract)	Vascular dementia mouse model	Hippocampal CA1 protection; improved oxidative/endothelial markers	Moderate (single study)	Limited replication
Antidiabetic (lipid fraction)	II (Extract)	Alloxan-induced diabetic mice	↓ Blood glucose 80%; ↑ insulin; ↑ islet number (41.8% vs. 11.5%)	High (30-day study with histology)	Type 1 diabetes model; not T2DM
Antidiabetic (lipid fraction)	II (Extract)	3T3-L1 adipocytes	↑ PPARγ mRNA 175%; ↑ GLUT4 mRNA 166.6%	High (molecular evidence)	In vitro; in vivo confirmation needed
Antithrombotic (UGP2A)	I (Isolated)	DVT mouse model	Inhibited thrombus formation; ↓ IL-6, TNF-α	High (purified compound)	IV administration; oral bioavailability unknown
Anti-inflammatory (UGP2A)	I (Isolated)	IL-1β-stimulated HUVECs	↓ vWF, P-selectin, IL-6, TNF-α; blocked NF-κB translocation	High (mechanistic)	Pathway partially dependent on TLR4/MyD88
Anti-inflammatory (UGP2A)	I (Isolated)	RAW264.7 macrophages	Inhibited iNOS/COX-2; blocked NF-κB p65 nuclear translocation	High (well-established model)	In vitro; in vivo macrophage effects not confirmed
Cytotoxicity (benzoquinones)	I (Isolated)	A549 cells	IC_50_ = 0.26 equivalent/mL; DNA damage (comet assay)	High (concentration-dependent)	Whole defensive secretion; individual quinone effects not tested
Genotoxicity (phenolic fractions)	I (Isolated fractions)	HaCaT keratinocytes	Concentration-dependent cytotoxicity and genotoxicity	Moderate (fraction not pure compound)	Pure compounds not tested
Genomic basis	III (Genomic)	Chromosome-level genome assembly	Identified SOD, CAT, GST, HSP, Laccase2 gene homologs	Moderate (homology-based)	Gene expression and protein function not validated
Molecular docking	III (Computational)	CB-Dock2 predictions	Hypothetical binding modes with SOD, TLR4, NF-κB, PPARγ, Keap1	Low (hypothesis-generating)	Requires experimental validation
Human safety	IV (Case reports)	Human ingestion	Colitis, acute eosinophilic pneumonia, palpable purpura	Moderate (rare events)	Uncontrolled; confounding factors
Human safety	IV (Case reports)	Experimental infection	H. diminuta cysticercoid development (92%)	Moderate (experimental)	Laboratory conditions; farming impact unknown
Plastic degradation	II (Whole organism)	PS, PE, PP degradation experiments	Larvae degrade plastics; gut microbiome changes	High (multiple studies)	Mechanism (host vs. microbiome) not fully resolved

* Confidence Rating: High = multiple studies/reproducible; Moderate = single or limited studies but consistent; Low = preliminary/correlational. ↑ indicates increase, ↓ indicates decrease.

## 5. Translational Challenges and Future Directions

The preceding sections have established that *Ulomoides dermestoides* possesses multi-target antioxidant and anti-inflammatory activities; however, most current studies remain at the level of crude extracts and animal models. The following discussion addresses feasible future directions from three dimensions—raw material quality control, safety risks, and industrial translation: taking into account documented real-world cases.

### 5.1. Raw Material Heterogeneity and Quality Control

*U. dermestoides* used across different laboratories varies substantially in developmental stage (adults or larvae), feed (wheat bran/peanuts/fruits and vegetables), geographic origin (China, Argentina, Brazil), and processing methods (freeze-drying/oven-drying, aqueous/alcohol extraction), directly leading to fluctuations in MBQ/EBQ content, fatty acid profiles, and protein composition. For instance, Jasso-Villagomez et al. reported a total lipid content of 24.7%, which differs markedly from the data of Zhou and Chen (approximately 18%), a discrepancy most likely attributable to dietary differences [12]. The chromosome-level genome released in 2024 (BioProject PRJNA1135335) provides a unified benchmark for precise species identification and functional gene screening [18]. Researchers have successfully identified multiple antioxidant proteins from the larval aqueous extract of *U. dermestoides* using LC-MS/MS and analyzed their gene homology [43]. That study also determined the catalase-specific activity of the aqueous extract to be approximately 45.9 μmol/min/mg protein, offering a clear paradigm for establishing correlations between enzymatic activity and chemical composition. Future efforts should build upon this foundation to construct a more comprehensive quality control model.

A case worth referencing is the systematic characterization of *U. dermestoides* defensive volatiles by Villaverde et al. using HS-SPME coupled with GC-MS, in which eight compounds, including MBQ and EBQ, accounted for over 90% of the total volatile content [11]. This analytical approach can serve as a standard method for batch-to-batch consistency testing of raw materials. Furthermore, the key gene Laccase2, which is involved in the biosynthesis of benzoquinone-defensive substances, has been identified in *Tribolium castaneum* [52]. Homologs of this gene are also present in the *U. dermestoides* genome; in the future, qPCR detection of Laccase2 expression levels could be employed to evaluate the impact of insect stress status on the accumulation of bioactive substances. When the European Union approved yellow mealworm (*Tenebrio molitor*) as a novel food, detailed data on rearing, processing, and contaminant testing were required [79]; if *U. dermestoides* is to meet equivalent standards, at least three to five enterprises or research institutions would need to collaborate in formulating group standards.

### 5.2. Safety Assessment

Pending the availability of direct safety data for *U. dermestoides*, toxicological findings from closely related species can serve as a reference for evaluation.

Freeze-dried defatted larval powder of *Zophobas atratus* (giant mealworm, belonging to the same family Tenebrionidae as *U. dermestoides*) was administered to SD rats by gavage at doses of 1250, 2500, and 5000 mg/kg/day in a 13-week repeated-dose oral toxicity study. No toxicological alterations were observed in clinical signs, body weight, food consumption, hematology, clinical biochemistry, or histopathology, and no induction of serum IgE was detected. The no-observed-adverse-effect level (NOAEL) was established at 5000 mg/kg/day [77]. Another 13-week oral toxicity study on *Locusta migratoria* likewise reported no adverse effects at a dose of 3000 mg/kg/day [80]. Although these data cannot be directly equated to safety conclusions for *U. dermestoides*, they at least indicate that, under appropriate rearing and processing conditions, tenebrionid insects possess a relatively high safety margin as food/feed ingredients.

With regard to genotoxicity assessment, Crespo et al. have clearly demonstrated that MBQ and EBQ in the defensive secretion of *U. dermestoides* exhibit concentration-dependent cytotoxicity and DNA damage in both A549 cells and normal monocytes [10]. However, these toxicity data pertain to the whole-insect defensive secretion and do not imply equivalent toxicity for isolated and purified lipid fractions, protein hydrolysates, or the UGP2A glycoprotein. These separated fractions have shown good tolerability in existing experiments [12,21]. Therefore, safety evaluation should adopt a fractionation-based, stepwise assessment: initially, the lipid fraction and protein hydrolysates should be subjected to the Ames test and the mouse bone marrow micronucleus test; for UGP2A, hemolysis and tail bleeding assays for which preliminary data are already favorable should be conducted; and only after non-toxicity is confirmed should studies be advanced to 13-week repeated-dose toxicity tests.

Regarding allergenicity, research on *T. molitor* has clearly demonstrated that tropomyosin is the major IgE cross-reactive allergen in insect proteins, sharing high homology with crustacean allergens; serum IgE from patients allergic to dust mites and crustaceans can functionally activate basophils [78]. The immunoglobulin E (IgE)-binding capacity of *T. molitor* proteins remains detectable after thermal processing (boiling) and is significantly reduced only in fried samples. This cross-reactivity has been confirmed in the EFSA assessment report: *T. molitor* proteins may elicit allergic reactions in individuals allergic to crustaceans, dust mites, and mollusks. Given that *U. dermestoides* and *T. molitor* belong to the same family Tenebrionidae, the allergen repertoire of *U. dermestoides* proteins should be systematically evaluated to determine sequence homology with tropomyosin and IgE cross-reactivity, and the effects of thermal processing or enzymatic hydrolysis on allergenicity should be explored [81,82].

### 5.3. Pathways to Industrialization

The industrialization of *U. dermestoides* should proceed in a stratified manner, with the following practical cases and research foundations available for reference.

Functional foods and feed: Flores et al. reported that the *U. dermestoides* protein hydrolysate exhibited ABTS radical scavenging activity of 104 μmol Trolox/g and produced inhibition zones (9–13.5 mm) against Proteus and Shigella [40]. *U. dermestoides* peptide powder could be developed following the model of *T. molitor* protein hydrolysates used as sports nutrition supplements.

With respect to formulation technology, Okagu et al. reported the construction of nanocomplexes using *T. molitor* protein–chitosan for encapsulating hydrophobic nutraceuticals. Curcumin was bound primarily through hydrophobic interactions to the hydrophobic core of the insect protein nanoparticles; the chitosan coating controlled the nanoparticle size at 143–178 nm with encapsulation efficiencies of 30–47%, and improved the release stability and thermal stability of curcumin under simulated oral conditions [83]. For *U. dermestoides* protein/peptide bioactive substances such as the UGP2A glycoprotein, this encapsulation strategy could be adapted to prepare nano-delivery systems via spray drying or electrospray techniques, thereby enhancing oral bioavailability and reducing degradation by pepsin/trypsin.

Benzoquinone residues in products must remain below the detection limit. In the closely related species *Alphitobius diaperinus*, Hassemer et al. have already quantified six quinones (MBQ, EBQ, *p*-benzoquinone, and three hydroquinones) in abdominal glands using GC-MS [84]. *U. dermestoides* could adopt this method to establish factory-release testing standards for benzoquinone residues and employ activated carbon adsorption or supercritical CO_2_ extraction for quality control.

Caballero-Gallardo et al. reported that rosemary and citronella essential oils achieved 100% repellency against *U. dermestoides* at 16 μL/mL, while the carvone chemotype of Lippia alba essential oil exhibited an RC_50_ as low as 1.5–2.2 μL/mL [16]. These essential oils could be developed as natural repellents for stored grain protection. An engineering challenge that needs to be addressed is the sustained-release microencapsulation of essential oils (e.g., using β-cyclodextrin embedding) to prolong their duration of efficacy. Citronella essential oil microcapsules have already been applied in stored grain protection, and *U. dermestoides* essential oil formulations could adopt this technology [85].

*U. dermestoides* larvae can degrade polystyrene (PS), polyethylene (PE), and polypropylene (PP), with gut microbiota playing a key role in plastic degradation [54,60]. The research system for plastic degradation by *T. molitor* is more mature; Wu et al. have described in detail the methodologies and procedures for characterizing plastic degradation by *T. molitor* larvae and their gut microbiota using PS as a substrate [86]. Antibiotic suppression experiments have confirmed that even when Gram-positive bacteria, Gram-negative bacteria, and fungi are inhibited, the larval gut retains the capacity to degrade PS, indicating that the host’s own enzymes (e.g., laccase, peroxidase) also play an independent role in degradation [86,87]. *U. dermestoides* research could follow this paradigm by employing antibiotic suppression experiments to distinguish the contributions of gut microbiota and host enzyme systems; using 16S rRNA sequencing to identify specific microbial community shifts induced by plastic substrates; and identifying host oxidoreductase genes associated with polymer depolymerization. At the industrial translation level, companies are already using *T. molitor* larvae to process polystyrene foam and subsequently extracting larval protein as animal feed. *U. dermestoides* could replicate this model, although the safety of the insects after plastic processing and the risk of contaminant accumulation must be assessed.

UGP2A has demonstrated antithrombotic activity in the DVT model with favorable in vitro safety profiles [21]. However, the oral bioavailability of glycoproteins is extremely low, and only intravenous administration data are currently available. Future studies should prepare UGP2A-loaded poly(lactic-co-glycolic acid) (PLGA) nanoparticles or liposomes, investigate their pharmacokinetic parameters (plasma half-life, Cmax, AUC) after oral administration, and evaluate their effects on prothrombin time (PT) and activated partial thromboplastin time (APTT). If oral efficacy can be demonstrated without prolonging bleeding time, UGP2A could enter preclinical development as a novel antithrombotic drug candidate. Table 6 summarizes the major translational challenges (raw material heterogeneity, safety concerns, formulation barriers) and corresponding strategies for industrial development of U. dermestoides-based products.

**Table 6:** Key translational bottlenecks and resolution pathways for *Ulomoides dermestoides*.

Bottleneck Category	Specific Problem	Proposed Solution	Technical Approach/Method	Reference Species/Technology Source	Existing Data Support	References
Raw material heterogeneity	MBQ/EBQ content varies by 5–10 fold between batches	Establish standardized feed formulation + fingerprint-based quality control	HS-SPME/GC-MS quantification; establish characteristic fingerprint	Volatilome study of *U. dermestoides* itself	MBQ/EBQ account for >90% of total volatiles and can serve as markers	[11]
Genotoxicity	Benzoquinones unequivocally cause DNA damage	Conduct stepwise toxicological assessment of isolated fractions	Ames test, mouse bone marrow micronucleus test	Whole-insect secretion of *U. dermestoides* (toxicity control); *Tenebrio molitor*/Zophobas (safety control)	IC_50_ of whole secretion = 0.26 equivalent/mL; lipid fraction exhibits good safety	[77,80]
Allergy risk	Cross-reactivity with allergens such as tropomyosin unknown	ELISA-based detection of IgE cross-reactivity + processing-induced allergen reduction	Serum IgE-binding inhibition assay; thermal processing (boiling, frying)	Allergen research on *T. molitor* (confirmed by EU EFSA)	*T. molitor* tropomyosin is highly homologous to crustacean allergens; thermal processing reduces allergenicity	[78]
Low oral bioavailability	Proteins/peptides are susceptible to degradation by gastric acid and enzymes	Encapsulation in nano-delivery systems	Chitosan nanoparticles; PLGA nanoparticles; spray drying/electrospray	*T. molitor* protein–chitosan encapsulation of curcumin technology	Encapsulated particle size 143–178 nm, encapsulation efficiency 30–47%, improved release stability	[83]
Short duration of repellent efficacy	Essential oils are highly volatile with short action time	Microencapsulated sustained-release formulation	β-Cyclodextrin embedding; indoor simulated stored-product efficacy trial	Citronella essential oil microcapsule technology for stored grain protection	Rosemary and citronella oils achieve 100% repellency against *U. dermestoides*; RC_50_ as low as 1.5–2.2 μL/mL	[16,83,88]
Safety of post-degradation insects	Potential accumulation of contaminants (heavy metals, plasticizers)	Test safety of post-degradation insects + isolate degrading enzymes	GC-MS/MS for contaminant detection; host enzyme isolation (laccase, peroxidase)	Research on plastic degradation mechanisms of *T. molitor* (antibiotic suppression experiments confirm host enzyme contribution)	*U. dermestoides* degrades PS, PE, PP; synergy between gut microbiota and host enzymes	[54,60,86,87]
Lack of quality control methods	Absence of standardized analytical procedures	Establish a multi-tier QC system (chemical composition–enzyme activity–gene expression)	HS-SPME/GC-MS fingerprint; LC-MS/MS proteomics; qPCR	Research on *U. dermestoides* itself (volatilome, proteome, genome)	CAT-specific activity ~45.9 μmol/min/mg protein; Laccase2 gene homolog exists	[11,43,52]

## 6. Conclusions

*Ulomoides dermestoides*, a tenebrionid beetle with a history of traditional medicinal use and a growing body of modern research, has garnered multi-level experimental evidence supporting its antioxidant and anti-inflammatory potential. Chemical analyses have demonstrated that its volatile defensive secretions are dominated by MBQ and EBQ, which together account for over 90% of total volatiles, and are also rich in unsaturated fatty acids such as oleic acid and linoleic acid, along with various phenolic and terpenoid constituents. Proteomic and enzymatic activity studies have further identified the presence of antioxidant and stress protein complexes, including SOD, CAT, peroxiredoxin (PRX), GST, and heat shock proteins (HSP60/HSP70), in the aqueous extract; its CAT-specific activity reaches 45.9 μmol/min/mg protein, and the extract extends lifespan and confers resistance against paraquat-induced oxidative damage in a *Caenorhabditis elegans* model. The chromosome-level genome assembly completed in 2024 has provided a genetic foundation for functional gene mining, while the glycoprotein UGP2A isolated and characterized in 2026 inhibits NF-κB activation via the TLR4/MyD88 pathway and attenuates endothelial inflammation and thrombosis, thereby offering a pathway-level molecular explanation for the blood-activating property of *U. dermestoides*.

Importantly, *U. dermestoides* is not currently a clinically validated therapeutic modality. Most pharmacological studies rely on crude extracts or whole-insect powders, leaving the target specificity and dose–response relationships of bioactive components largely undefined; benzoquinone- and phenol-containing crude preparations have been unequivocally shown to exhibit cytotoxicity and genotoxicity, and human case reports have documented adverse reactions including colitis, acute eosinophilic pneumonia, and purpura. Although safety evaluations of closely related species, such as a no-observed-adverse-effect level (NOAEL) of 5000 mg/kg/day for *Zophobas atratus*, provide a reference framework for tenebrionid insects, subchronic and reproductive toxicity data for *U. dermestoides* itself remain lacking. Furthermore, issues such as raw material heterogeneity, the absence of quality markers, and the low oral bioavailability of proteinaceous bioactive components severely constrain the industrialization process.

It is essential to emphasize that the current evidence, while promising, remains preclinical in nature. The absence of standardized extracts, defined quality markers, validated active ingredients, pharmacokinetic data, and clinical safety information means that *U. dermestoides* preparations should not be considered ready for human therapeutic use. The pathway from traditional use to evidence-based medicine requires systematic, phased investigation from chemical characterization to preclinical toxicology to controlled clinical trials. This review is intended to provide a conceptual roadmap for such development, not to advocate for clinical application at the present stage.

Future research should prioritize establishing a volatilome fingerprint using HS-SPME/GC-MS for quality control and defining safety thresholds for hazardous components such as MBQ and EBQ. Bioassay-guided fractionation is needed to isolate and identify individual active components, followed by standardized toxicological screening (Ames test, micronucleus assay, acute oral toxicity) for the most promising fractions. A multi-tier quality control system linking chemical composition, enzyme activity, and gene expression should be developed and validated. Subchronic and reproductive toxicity studies in rodents are required to establish NOAEL and determine target organs of toxicity. The oral bioavailability of lead compounds should be investigated using nano-delivery systems with characterization of PK parameters, and allergenicity should be evaluated through IgE-binding assays. Ultimately, promising candidates should be advanced into Phase I clinical trials, supported by GMP-compliant production processes for standardized extracts with defined quality markers, leading to controlled clinical trials for specific indications based on the strongest preclinical evidence.

Looking forward, the development of *U. dermestoides* should advance from pharmacodynamic studies of crude extracts toward component isolation, target validation, and formulation optimization. In the near term, efforts should focus on establishing a volatilome fingerprint based on HS-SPME/GC-MS and a proteomic quality control system based on LC-MS/MS, defining safety thresholds for hazardous components such as MBQ and EBQ, and developing functional foods from protein hydrolysates and essential oil microcapsule repellents; in the long term, oral delivery system research and rigorous toxicological evaluation of lead compounds such as UGP2A must be completed. In summary, *U. dermestoides* is not yet a clinically validated therapeutic modality; however, as an insect pharmacological resource for discovering bioactive substances with antioxidant, anti-inflammatory, metabolic regulatory, and vascular protective activities, it possesses considerable scientific value and translational potential that should not be overlooked.

## Data Availability

No new data were created or analyzed in this study. Data sharing is not applicable to this article.
